# Knockout of Mpv17-Like Protein (M-LPH) Gene in Human Hepatoma Cells Results in Impairment of mtDNA Integrity through Reduction of TFAM, OGG1, and LIG3 at the Protein Levels

**DOI:** 10.1155/2018/6956414

**Published:** 2018-09-17

**Authors:** Reiko Iida, Misuzu Ueki, Toshihiro Yasuda

**Affiliations:** ^1^Division of Life Science, Faculty of Medical Sciences, University of Fukui, Fukui 910-1193, Japan; ^2^Organization for Life Science Advancement Programs, Faculty of Medical Sciences, University of Fukui, Fukui 910-1193, Japan; ^3^Division of Medical Genetics and Biochemistry, Faculty of Medical Sciences, University of Fukui, Fukui 910-1193, Japan

## Abstract

Human Mpv17-like protein (M-LPH) has been suggested to participate in prevention of mitochondrial dysfunction caused by mitochondrial DNA (mtDNA) damage. To clarify the molecular mechanism of M-LPH function, we knocked out M-LPH in human hepatoma HepG2 using CRISPR-Cas9 technology. An increase in mtDNA damage in M-LPH-KO HepG2 cells was demonstrated by PCR-based quantitation and 8-hydroxy-2′-deoxyguanosine (8-OHdG) measurement. Furthermore, confocal immunofluorescence analysis and Western blot analysis of mitochondrial extracts demonstrated that M-LPH-KO caused reductions in the protein levels of mitochondrial transcription factor A (TFAM), an essential factor for transcription and maintenance of mtDNA, and two DNA repair enzymes, 8-oxoguanine DNA glycosylase (OGG1) and DNA ligase 3 (LIG3), both involved in mitochondrial base excision repair (BER). Accordingly, it was suggested that the increase in mtDNA damage was due to a cumulative effect of mtDNA instability resulting from deficiencies of TFAM and diminished ability for BER arising from deficiencies in BER-related enzymes. These findings suggest that M-LPH could be involved in the maintenance of mtDNA, and therefore mitochondrial function, by protecting proteins essential for mtDNA stability and maintenance, in an integrated manner.

## 1. Introduction

Mitochondria are vital organelles in eukaryotic cells involved in essential functions such as energy production through oxidative phosphorylation (OXPHOS), maintenance of calcium homeostasis, regulation of apoptosis and necrosis, lipid metabolism, and immune responses [1]. Mitochondria possess their own double-stranded circular DNA (mtDNA), which encodes 22 tRNAs, 2 rRNAs, and 13 subunits of the OXPHOS complexes. Because of constant attack by reactive oxygen species (ROS), generated as by-products of oxidative metabolism, mtDNA has a much higher frequency of mutations than nuclear DNA (nDNA) [2–4]. mtDNA is organized as DNA-protein complexes called nucleoids, which are covalently associated with the inner mitochondrial membrane [5], and more than 50 nucleoid-associated proteins play roles in mtDNA maintenance and gene expression [6]. Mitochondrial transcription factor A (TFAM), a member of the high-mobility group (HMG)- box family protein, is one of the major components of nucleoids. TFAM plays an essential role in transcription and maintenance of mtDNA and packages mtDNA by nonspecific binding and multimerization [7, 8], thus protecting mtDNA from damage by ROS [9, 10]. Meanwhile, it has been revealed that mitochondria possess several repair systems, including base excision repair (BER), mismatch repair, and direct reversal [11]. The mechanism of BER in mitochondria is similar to that in the nucleus, and many of the BER enzymes working in the nucleus, including 8-oxoguanine DNA glycosylase (OGG1) and DNA ligase 3 (LIG3), have also been identified in mitochondria. There is evidence that BER proteins in mitochondria are localized to the inner membrane and thus to the nucleoid [12]. However, details of the mechanism for regulation of these proteins involved in the maintenance of mtDNA integrity are not fully understood.

Mpv17-like protein (M-LP, synonym: Mpv17L) belongs to the Mpv17/PMP22 protein family. M-LP was initially identified as a product of an age-dependently expressed gene in mouse kidney [13–15], and subsequently the human ortholog of M-LP (M-LPH) was identified [16]. Expression of the *M-LPH* gene is regulated by a transcriptional repressor, ZNF205/RhitH (human regulator of heat-induced transcription) [17, 18], and expression of the *ZNF205/RhitH* gene is in turn regulated by the GA-binding protein, one of the key transcriptional regulators of the mitochondrial electron transport system [19]. Recent coimmunoprecipitation experiments have revealed that M-LPH interacts with the H2A histone family, member X (H2AX), ribosomal protein S14, ribosomal protein S3, and B-cell receptor-associated protein 31 [20]. These proteins are known to be closely correlated with the DNA damage response, endoplasmic reticulum stress, DNA repair, apoptosis, or mitochondrial fission. Although the molecular function of M-LPH has not been clarified, a number of our findings obtained so far strongly suggest that M-LPH is involved in the maintenance of mtDNA, therefore protecting cells from mitochondrial dysfunction: (i) overexpression of M-LPH in MCF-7 breast cancer cells reduces the generation of intracellular ROS and loss of mitochondrial membrane potential (ΔΨ_m_) caused by an inhibitor of the respiratory chain [19]. (ii) Knockdown of M-LPH in HK-2 normal kidney cells leads to an increase in mtDNA damage and reduces the expression of mtDNA-encoded genes [20]. (iii) In HK-2 cells, M-LPH is colocalized with mitochondrial BER enzymes, LIG3, and DNA polymerase *γ* under oxidative stress [20]. In order to clarify the molecular mechanism of M-LPH function, we first tried to generate M-LPH-knockout (KO) HK-2 cells using CRISPR-Cas9 technology. However, all single-cell clones obtained did not proliferate and gradually died within two months, suggesting a possibility that knockout of the M-LPH gene was lethal to HK-2 cells. We next chose HepG2 hepatoma cells which express a moderate level of M-LPH and successfully knocked out M-LPH in HepG2 cells. In the present study, we confirmed that lack of M-LPH resulted in an increase in mtDNA damage. Furthermore, we found that M-LPH-KO resulted in reductions of mitochondrial TFAM, OGG1, and LIG3 at the protein level. These observations suggested that an increased degree of mtDNA damage in M-LPH-deficient cells was attributable to the cumulative effect of two factors: instability of mtDNA due to lack of TFAM and diminished capability for mtDNA damage repair resulting from lack of BER enzymes.

## 2. Materials and Methods

### 2.1. Cells

The human hepatoma cell line HepG2 was obtained from the Japanese Collection of Research Bioresources Cell Bank (JCRB, Osaka, Japan). The cells were maintained in EMEM medium supplemented with 10% fetal calf serum, 0.29 mg/ml L-glutamine, 0.11 mg/ml sodium pyruvate, 0.1 mM nonessential amino acids, and 1.5 mg/ml sodium bicarbonate, in an atmosphere of 5% CO_2_ at 37°C.

### 2.2. CRISPR/Cas9-Mediated Knockout of M-LPH

CRISPR sequences targeting exon 1 of the human *M-LPH* gene were designed using the CRISPR design tool (http://crispr.mit.edu/), and the corresponding oligonucleotides were synthesized: 5′-CACCCGAGCCGTAAAGCAGCACGT-3′ and 5′-AAACACGTGCTGCTTTACGGCTCG-3′. The pair of oligonucleotides encoding the 20 nt guide sequences were annealed and cloned into the *Bbs*I sites of pX459 (Addgene plasmid ID: 48139) and transfected into HepG2 cells using Lipofectamine 3000 (Invitrogen, Carlsbad, CA). Transfected cells were selected with 1.5 *μ*g/ml puromycin. Single-cell clones were evaluated for insertions/deletions (indels) in the target locus using the Surveyor Mutation Detection kit (Integrated DNA Technologies Inc., Coralville, IA). Next, the genomic region including the target locus was PCR-amplified, and the PCR products obtained were subcloned into the pCR2.1 vector (Invitrogen) and sequenced to confirm the presence of indels in the target sequence of each allele. Finally, elimination of M-LPH expression at the protein level was confirmed by Western blot analysis.

### 2.3. Cell Growth Assay

To measure the proliferation capability of M-LPH-wild-type (WT) and -KO cells, 6.7 × 10^4^ cells were seeded onto 12-well plates (*n* = 3 each) and then incubated for 24, 48, and 72 hours. Cell counts were determined using a hemocytometer, and cell doubling times were calculated using software available on the Web (http://www.doubling-time.com/compute.php).

### 2.4. mtDNA Damage Assessment

Mitochondria were isolated from M-LPH-WT and -KO cells using a mitochondria isolation kit (Thermo Scientific, Waltham, MA). Isolation of mtDNA was carried out according to the method described by Goo et al. [21]. Assessment of mtDNA damage was performed by quantitative-PCR (Q-PCR) as described previously [20, 22]. Two sets of primers were for the sequences encoding ATP synthase 6 (MTATP6) and ATP synthase 8 (MTATP8), both of which are localized in the hot regions sensitive to free-radical attack [23], and another two sets of primers were designed for the D-loop regions (positions 368–476 and 16,457–16,535 of NC_012920), where lesions are rarely observed. The degree of mtDNA damage was calculated by dividing the relative value of the PCR product by that amplified from the mitochondrially encoded 16S RNA (*MTRNR2*) region, where lesions are rarely observed [22]. All of the primer sequences used in this study are summarized in Table 1.

siRNA-mediated knockdown of M-LPH was carried out as described previously [19]. M-LPH-WT cells were transfected with control siRNA or siRNA for knockdown of M-LPH, respectively.

### 2.5. Q-PCR Analysis

Total RNA was extracted with an RNeasy Mini kit (Qiagen, Chatsworth, CA), and cDNA was synthesized using a PrimeScript RT reagent kit with gDNA Eraser (Takara, Bio Inc., Shiga, Japan). Q-PCR was performed using the StepOnePlus real-time PCR System (Applied Biosystems, Foster city, CA) and the Power SYBR Green Master Mix (Applied Biosystems) in accordance with the manufacturer's instructions. For assessment of the mRNA levels per single cell, the expression of each gene was normalized against that of *β*-actin, whereas those per single mtDNA molecule were assessed by normalization against MTRNR2 expression. Measurement of transcripts encoding the mitochondrial form of OGG1 and LIG3 was carried out using primers purchased from Qiagen (OGG1, QT01863330; LIG3, and QT00017115). The mtDNA content was determined using a TaqMan probe specific for the tRNA^leu^ region, and *β*2-microglobulin was used as a nuclear gene for normalization in accordance with the previous paper [24]. All PCR assays were performed at least three times.

### 2.6. Measurement of Mitochondrial ΔΨ_m_, ROS Level, ATP Level, and 8-Hydroxy-2′-deoxyguanosine (8-OHdG) Level

Loss of mitochondrial ΔΨ_m_ was monitored using a Cell Meter JC-10 Mitochondrial Membrane Potential Assay kit (AAT Bioquest, Sunnyvale, CA). M-LPH-WT and -KO cells were grown in 96-well plates (*n* = 5 each) and stained with a fluorescent cationic dye, JC-10, in accordance with the manufacturer's instructions. The fluorescence intensities for the monomeric forms and aggregates of JC-10 were monitored at Ex/Em = 485/538 nm and Ex/Em = 544/590 nm using a SpectraMax M5 microplate reader (Molecular Device, Sunnyvale, CA). The ratio of the fluorescence intensities at Em 538/590 was used for data analysis.

Intracellular ROS levels were assessed by the 2′,7′-dichlorofluorescein diacetate (DCF-DA) fluorescence method [25]. Briefly, M-LPH-WT and -KO cells were each grown in 60 mm dishes to 70–80% confluency (*n* = 4 each) and incubated with 10 *μ*M DCF-DA for 1 hour. The cells were then washed twice with PBS and sonicated using a Bioruptor UCD-200 (Diagenode, Seraing, Belgium) with 10 10 sec bursts in an ice water bath. Then, cell lysate was collected and the DCF fluorescence was immediately measured in a 96-well plate at Ex/Em = 485/535 nm. Intracellular ROS levels were also measured by staining with MitoSOX Red (Invitrogen). M-LPH-WT and -KO cells were each grown in 6-well plates to 70–80% confluency and incubated with 5 *μ*M MitoSOX for 15 min. The cells were then trypsinized, washed with PBS containing 0.1% BSA, suspended in 500 *μ*l of PBS containing 0.01% BSA, and analyzed using a fluorescence-activated cell sorter, BD FACSCanto II (BD Biosciences, San Jose, CA). MitoSOX Red was exposed to laser irradiation at 488 nm, and the data were collected at the 585/42 nm (FL2) channel.

Intracellular ATP levels were measured using an intracellular ATP Assay kit (TOYO B-Net, Tokyo, Japan) according to the manufacturer's instruction. Briefly, the same number of M-LPH-WT and -KO cells (*n* = 5 each) was lysed by addition of the ATP extraction reagent (1.5 × 10 ^3^ cells per 150 *μ*l). After centrifugation, 100 *μ*l supernatant and 100 *μ*l ATP detection reagent were mixed together in the dark. Luminescence was measured using a microplate reader. The levels of ATP were determined using the standard curve generated from known amounts of ATP.

For measurement of 8-OHdG, total DNA was extracted from M-LPH-WT and -KO cells using a QIAamp DNA Mini Kit (Qiagen). Levels of 8-OHdG in DNA samples were determined using an OxiSelect Oxidative DNA Damage ELISA Kit (Cell Biolabs Inc., San Diego, CA) in accordance with the manufacturer's instructions.

### 2.7. Western Blot Analysis

Nuclear and mitochondrial extracts were prepared using a Subcellular Protein Fractionation Kit for cultured cells (Thermo Scientific). SDS-PAGE electrophoresis and Western blot analysis were performed as described previously [26] using anti-M-LPH (HPA041180) and anti-LIG3 (HPA006723) antibodies purchased from Atlas Antibodies (Stockholm, Sweden), anti-voltage-dependent anion channel (VDAC, 55249-1-AP), anti-histone deacetylase 1 (HDAC1, 10197-1-AP), anti-histone cluster 1 H1 family member C (HIST1H1C, 15446-1-AP), and anti-Lon protease-like protein (LONP1, 15440-1-AP) antibodies from Proteintech (Chicago, IL), anti-TFAM (#8076) and anti-DNA polymerase *γ*, catalytic subunit (POLG, #13609) antibodies from Cell Signaling Technology (Danvers, MA), anti-OGG1 antibody (NB100-106) from Novus Biologicals (Littleton, CO), anti-heat shock protein 90 alpha family class A member 1 (HSP90AA1, GTX109753) and anti-gamma H2A histone family member X (*γ*-H2AX, GTX11174) antibodies from GeneTex (Irvine, CA), anti-Twinkle helicase (TWNK, ab83329) antibody from Abcam (Cambridge, UK), and anti-*β*-actin antibody (A2066) from Sigma (St. Louis, MO). Horseradish peroxidase-conjugated anti-rabbit IgG antibody was purchased from Thermo Scientific. Relative levels of expression of each protein were quantified by densitometric measurement. The gel profiles were obtained with the Multi Gauge software (Fujifilm, Tokyo, Japan). For examination of the phosphorylation status of mitochondrial proteins, we performed Zn^2+^-Phos-tag SDS-PAGE [27]. Zn^2+^-Phos-tag gels (8% polyacrylamide, 50 *μ*M Phos-tag) were prepared following the manufacturer's instruction (Wako Pure Chemical Industries, Osaka, Japan), and electrophoresis was performed at a constant current of 30 mA for 80 min (running buffer: 0.1 M Tris, 0.1 M MOPS, 0.1% (*w*/*v*) SDS, and 0.05 M sodium bisulfite pH 7.8). For *λ* protein phosphatase treatment, mitochondrial extracts from M-LPH-WT and -KO cells were incubated with or without 40 U/*μ*l of *λ* protein phosphatase (New England Biolabs, Ipswich, MA). Western blotting using Zn^2+^-Phos-tag gels was carried out in the same way, except that the gels were soaked three times for 10 min in blotting buffer containing 10 mM EDTA prior to transfer onto a PVDF membrane.

### 2.8. Colocalization Analysis

M-LPH-WT and -KO cells were grown for 1 day on collagen I-coated cover glasses (Asahi Glass, Tokyo, Japan). For mitochondrial staining, the cells were treated with 300 nM MitoTracker dye (CMXRos, Molecular Probes, Eugene, OR) in the culture medium for 30 min at 37°C. After washing with PBS, the cells were fixed with 4% paraformaldehyde in PBS for 15 min, permeabilized with 0.5% Triton X-100 in PBS for 5 min, and then treated with blocking reagent (Image-iT FX signal enhancer, Molecular Probes, Eugene, OR) for 30 min. The cells were immunostained using antibodies against TFAM, TWNK and *γ*-H2AX, and secondary antibodies corresponding to each primary antibody (Alexa Fluor 488 plus goat anti-rabbit and Alexa Fluor 647 plus goat anti-mouse antibodies, Molecular Probes). Antibodies were diluted in antibody diluents (Dako, Glostrup, Denmark) and incubated with cells overnight at 4°C. Cells were finally mounted in antifade reagent (SlowFade Diamond Antifade Mountant with DAPI, Molecular Probes), and fluorescence images were analyzed using a laser scanning confocal microscope (FV1200, Olympus, Tokyo, Japan).

### 2.9. Statistical Analysis

All of the assays were performed at least 3 times, and the results were presented as mean ± SD. Statistical analyses were performed by Student's *t*-test, at a significance level of *p* < 0.05.

## 3. Results

### 3.1. Generation of M-LPH-KO Cells Using the CRISPR/Cas9 System

**Figure 1:**
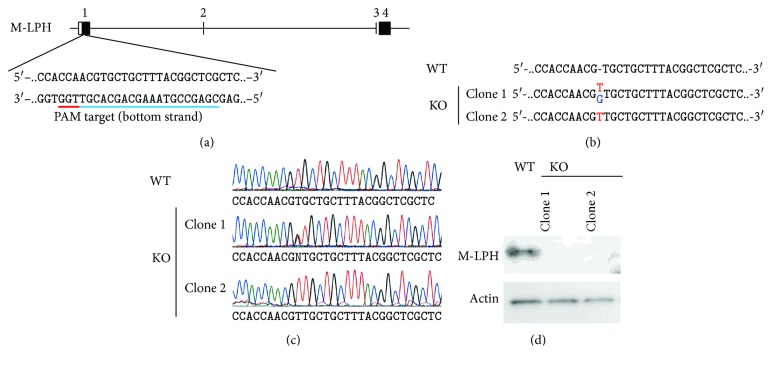
Generation of M-LPH-KO HepG2 cells using the CRISPR/Cas9 system. (a) The sgRNA was designed to target 20 pairs of bases in exon 1 of the *M-LPH* gene. (b) Sequences of the WT and two knockout (KO) clones. Clone 1 had one allele with a single T insertion while the other had a single G insertion. Clone 2 was homozygous for a single T insertion. (c) Results of sequence analysis of the wild-type (WT) and two established clones. (d) Western blot analysis of M-LPH was performed using cell extracts obtained from the WT and established clones. *β*-Actin was used as a loading control.

We designed a sgRNA that targeted 20 pairs of bases in exon 1 of the *M-LPH* gene as shown in Figure 1(a) and transfected the gRNA-Cas9 expression vector into HepG2 cells. Two single-cell clones were established by serial dilution in the presence of puromycin, and insertions in the targeted region of each allele were confirmed by sequencing analysis (Figures 1(b) and 1(c)). Clone 1 was heterozygous for a single T and a single G insertion, whereas clone 2 was homozygous for a single T insertion. Finally, the absence of M-LPH protein was verified by Western blot analysis (Figure 1(d)). Hereafter, representative results obtained using clone 1 are shown, as a series of experiments using both clones gave similar results.

### 3.2. Subcellular Localization of M-LPH in HepG2 Cells

**Figure 2:**
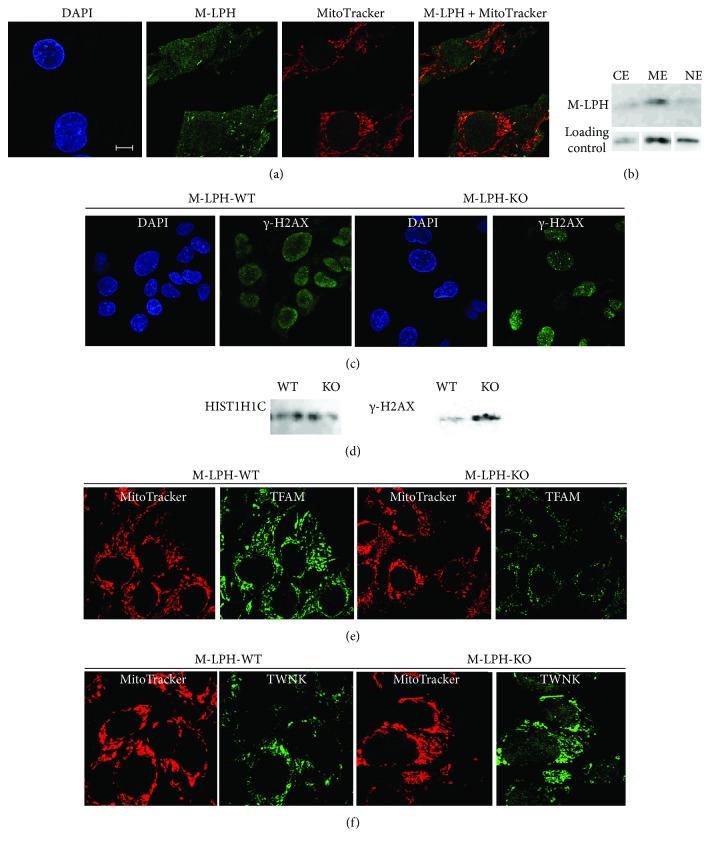
Confocal immunofluorescence and Western blotting analyses of M-LPH-WT and -KO HepG2 cells using specific antibodies. (a) In M-LPH-WT cells, the anti-M-LPH antibody displayed faint nuclear and cytosolic staining, and a small number of punctate staining foci were observed within the cytoplasm. All of these foci were colocalized with a subset of mitochondria. DAPI and MitoTracker were used for nuclear and mitochondrial staining, respectively. Scale bar: 10 *μ*m. (b) Subcellular fractionation/immunoblotting analysis of M-LPH-WT cells using specific antibodies against M-LPH. CE: cytosolic extract; ME: membrane extract; NE: nuclear soluble extract. Loading controls for CE, ME, and NE were Hsp90AA1, VDAC, and HDAC1, respectively. (c) M-LPH-KO resulted in a significant increase of *γ-*H2AX formation. (d) Western blot analysis of chromatin-bound extracts from MLPH-WT and -KO HepG2 cells. HIST1H1C was used as a loading control for nuclear protein. (e, f) Immunofluorescence detection showed that the protein level of TFAM was downregulated by M-LPH-KO (e), whereas that of TWNK was upregulated (f).

In our previous study using HK-2 cells, we found that M-LPH was localized predominantly in the nucleus, to some extent in the mitochondria, and marginally in the cytosol. To examine M-LPH localization in HepG2 cells, we performed confocal immunofluorescence analysis and subcellular fractionation, followed by immunoblotting analysis. The anti-M-LPH antibody revealed faint nuclear and cytosolic staining, and a small number of punctate staining foci were observed within the cytoplasm. All of these foci were colocalized with a subset of mitochondria, as demonstrated by MitoTracker conterstaining, indicating that M-LPH is localized in mitochondria (Figure 2(a)). Further examination using subcellular fractionation revealed that M-LPH was localized modestly in the membrane extract, and slightly in the nuclear and cytosolic extracts (Figure 2(b)), being consistent with the results of immunofluorescence experiments.

### 3.3. M-LPH-KO Reduces the Growth Rate of HepG2 Cells

**Figure 3:**
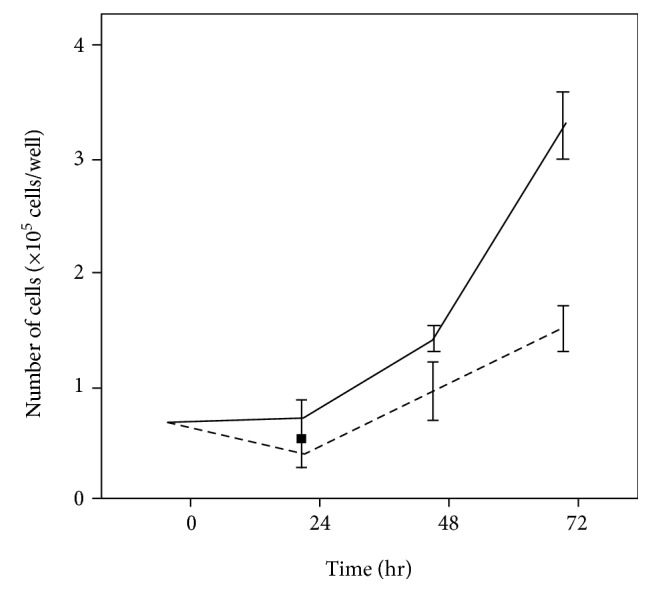
Effect of M-LPH-KO on the growth rate of HepG2 cells. The same number of M-LPH-WT and -KO cells was cultured in 6-well plates (*n* = 3 each), and the number of cells in each well was counted every 24 hours. The solid line and dashed line show the growth curves for M-LPH-WT and -KO cells, respectively. The bars represent the mean ± SD of the results from three independent experiments. The doubling time for M-LPH-KO cells (63.2 ± 9.84 h) were significantly longer than that for M-LPH-WT cells (31.8 ± 4.33 h) (*p* = 0.0072).

To ascertain whether M-LPH-KO has a physiological effect on HepG2 cell function, we first analyzed the growth rates of M-LPH-WT and -KO cells. As shown in Figure 3, the growth rate of M-LPH-KO cells was significantly lower than that of M-LPH-WT cells. The doubling times for M-LPH-WT and M-LPH-KO cells were 31.8 ± 4.33 and 63.2 ± 9.84 h, respectively (*p* = 0.0072).

### 3.4. M-LPH-KO Increases the Level of Damage in Nuclear and Mitochondrial DNA

In order to investigate whether reduction of cell growth is attributable to DNA damage, we analyzed the expression of *γ*-H2AX, a sensitive marker of nuclear DNA damage and repair, using immunofluorescence microscopy and Western blot analysis. The number of *γ*-H2AX foci was significantly increased by M-LPH-KO (Figure 2(c)), and much more amount of *γ*-H2AX protein was detected in nuclear extracts obtained from M-LPH-KO cells (Figure 2(d)). These results clearly showed that nDNA damage had occurred in M-LPH-KO cells.

**Figure 4:**
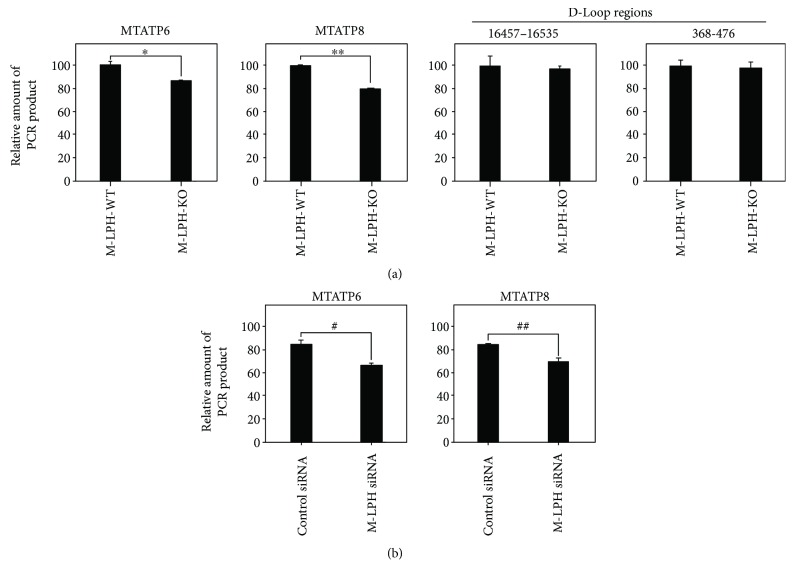
PCR-based mtDNA damage assessment. (a) M-LPH-WT and -KO HepG2 cells. The results are expressed as ratios relative to the value for M-LPH-WT cells. (b) M-LPH-WT cells transfected with control siRNA and those with M-LPH siRNA. The results are expressed as ratios relative to the value for M-LPH-WT cells transfected with control siRNA. MTATP6 and MTATP8 are localized in the hot regions sensitive to free-radical attack, whereas the two D-loop regions (positions 368–476 and 16,457–16,535 of NC_012920) are localized in the region where damage is rarely observed. The degree of mtDNA damage was calculated by dividing the relative value of the PCR product by that amplified from the MTRNR2 region. The bars represent the mean ± SD of the results from three independent experiments. Differences at *p* < 0.05 were considered to be statistically significant (^∗^*p* = 0.014, ^∗∗^*p* = 0.00028, ^#^*p* = 0.047, and ^##^*p* = 0.043).

**Figure 5:**
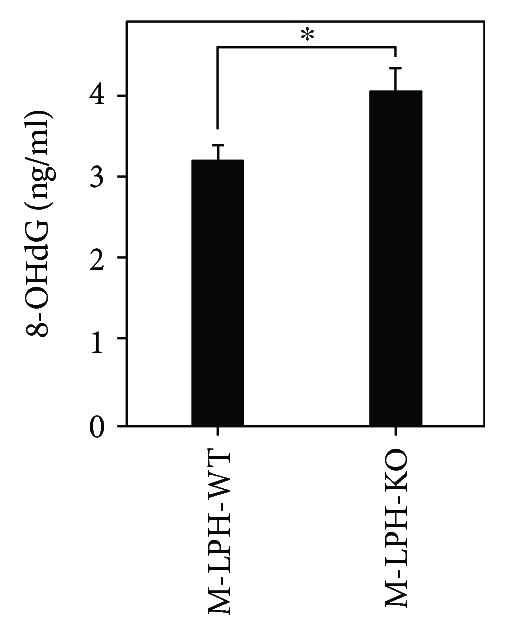
Measurement of the intracellular 8-OHdG level for M-LPH-WT and -KO HepG2 cells. The bars represent the mean ± SD of the results from four independent experiments. Differences at *p* < 0.05 were considered to be statistically significant (^∗^*p* = 0.0024).

We then examined mtDNA damage by quantifying the relative amount of PCR product amplified from 4 regions in mtDNA: two hot regions sensitive to free-radical attack (*MTATP6*, *MTATP8*) and two D-loop regions where damage is rarely observed. The amount of PCR products amplified from the *MTATP6* and *MTATP8* regions were significantly reduced in M-LPH-KO cells, whereas no reduction was observed in those amplified from D-loop regions (Figure 4(a)). To further confirm these results, we performed M-LPH knockdown (KD) experiments in M-LPH-WT cells and then examined the mtDNA damage (Figures 4(b)). As expected, levels of mtDNA damage were increased by M-LPH-KD. Taken together, it was suggested that the levels of damage to mtDNA hot regions under normal condition were increased by M-LPH-KO; that is, it appeared likely that M-LPH attenuated mtDNA damage induced by ROS, which are constantly generated as a consequence of aerobic respiration. Subsequently, we assessed the intracellular level of 8-OHdG, one of the major DNA lesions formed as a result of oxidative attack, and found that it was significantly increased in M-LPH-KO cells (Figure 5). Considering that mtDNA is a primary cellular target for ROS and that the level of 8-OHdG is much higher (3–16-fold) in mtDNA than in nDNA [28], the increase in the level of 8-OHdG in M-LPH-KO cells was revealed to be due mainly to oxidative damage of mtDNA.

### 3.5. Lack of Definitive Signs of Mitochondrial Dysfunction in M-LPH-KO Cells

**Figure 6:**
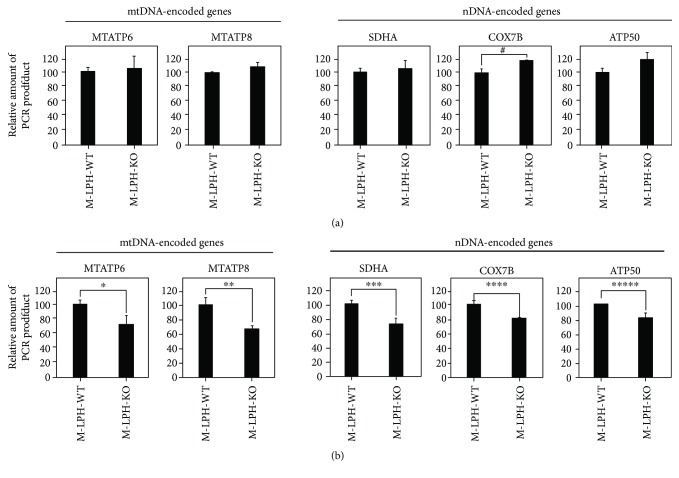
mRNA levels of nDNA- and mtDNA-encoded genes in M-LPH-WT and -KO HepG2 cells. (a) For assessment of the mRNA levels per single cell, the expression of each gene was normalized against that of *β*-actin. (b) For assessment of the mRNA levels per single mtDNA molecule, the expression of each gene was normalized against that of MTRNR2. The results are expressed as ratios relative to the value for M-LPH-WT cells. The bars represent the mean ± SD of the results from three independent experiments. Differences at *p* < 0.05 were considered to be statistically significant (^#^*p* = 0.0028, ^∗^*p* = 0.021, ^∗∗^*p* = 0.0050, ^∗∗∗^*p* = 0.0053, ^∗∗∗∗^*p* = 0.0021, and ^∗∗∗∗∗^*p* = 0.037). SDHA: succinate dehydrogenase complex flavoprotein subunit A; COX7B: cytochrome C oxidase subunit 7B; ATP50: ATP synthase, H+ transporting, mitochondrial F1 complex, O subunit.

**Figure 7:**
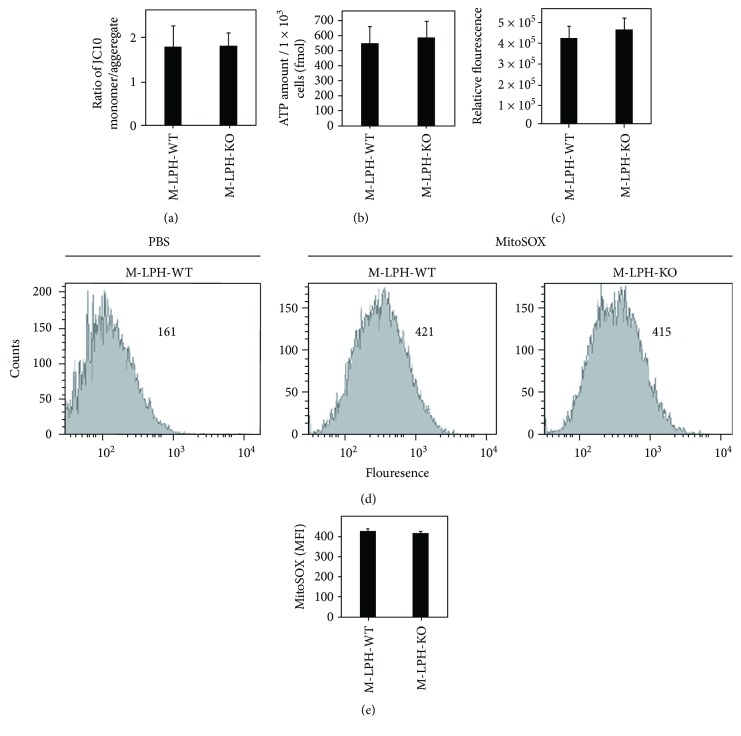
Measurement of intracellular ROS and ATP levels and mitochondrial ΔΨ_m_ in M-LPH-WT and -KO HepG2 cells. (a) Mitochondrial ΔΨ_m_ was monitored using a fluorescent cationic dye, JC-10. The fluorescence intensities for the aggregated and monomeric forms of JC-10 were monitored at Ex/Em = 485/538 nm and Ex/Em = 544/590, respectively. The ratio of fluorescence at 538 nm/590 nm was used for data analysis. The bars represent the mean ± SD of the results of five experiments. (b) The levels of intracellular ATP were determined based on luciferase's requirement for ATP in producing light. Luminescence was measured using a microplate reader, and the levels of ATP were determined using the standard curve generated from known amounts of ATP. The bars represent the mean ± SD of the results of five experiments. (c) Intracellular ROS levels were assessed by the DCF-DA fluorescence method. DCF fluorescence was measured at Ex/Em = 485/535 nm. The bars represent the mean ± SD of the results of four experiments. (d) Intracellular ROS levels were measured by flow cytometry using MitoSOX Red. The data shown are representative of two independent experiments with similar results. Numbers in graphs represent the mean fluorescence intensity (MFI). (e) MFI of MitoSOX Red. There was no significant difference between M-LPH-WT and -KO cells. The bars represent the mean ± SD of the results from two independent experiments.

The results of our previous M-LPH-KD study using human normal kidney (HK-2) cells had suggested a high possibility that the increased mtDNA damage observed in mtDNA-encoded genes would lead to reductions in the levels of their mRNA. Therefore, we examined the mRNA levels of these genes, as well as those of several nDNA-encoded genes involved in OXPHOS. Unexpectedly, the results of Q-PCR analysis revealed that M-LPH-KO did not cause a decrease in the intracellular levels of mRNAs for all of the genes examined (Figure 6(a)). In order to further examine the effect of MLPH-KO on mitochondrial function, we next analyzed intracellular ROS and ATP levels and mitochondrial ΔΨ_m_ (Figure 7). This revealed no significant differences between M-LPH-WT and -KO cells in any of the analyses performed, suggesting that the mitochondrial function of M-LPH-KO was not impaired, at least under normal conditions.

### 3.6. M-LPH-KO Increases the mtDNA Copy Number

**Figure 8:**
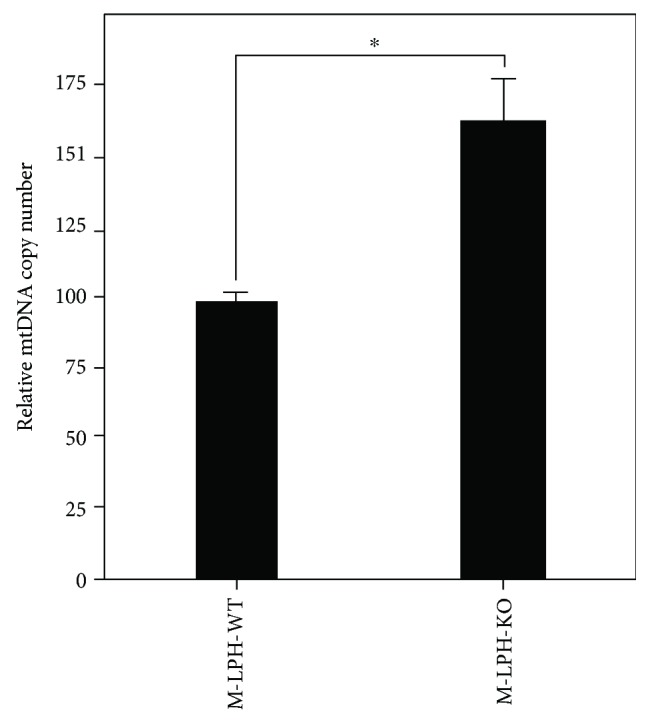
Analysis of the mtDNA content of M-LPH-WT and -KO HepG2 cells. mtDNA content was determined using the TaqMan probe assay. The results are expressed as ratios relative to the value for M-LPH-WT cells. The bars represent the mean ± SD of the results from three independent experiments. Differences at *p* < 0.05 were considered to be statistically significant (^∗^*p* = 0.0021).

Two observations of M-LPH-KO cells appeared to contradict each other: the increase in mtDNA damage (Figures 4(a) and 5) and the absence of mitochondrial dysfunction (Figures 6(a) and 7). As one possible explanation for this contradiction, we considered that mitochondrial dysfunction caused by mtDNA damage might be prevented by an increase in mtDNA copy number, as a number of studies have demonstrated the effectiveness of an increased mtDNA copy number for preservation of mitochondrial function [29, 30]. As we expected, mtDNA quantitation by Q-PCR revealed a copy number increase of 66% in M-LPH-KO cells compared with that in M-LPH-WT cells (Figure 8). Accordingly, the intracellular mRNA levels (i.e., mRNA levels per single cell) shown in Figure 6(a) appears to reflect the increase in the copy number of mtDNA in M-LPH-KO HepG2 cells. Moreover, when we evaluated mRNA levels per single mtDNA molecule by normalization against not *β*-actin but MTRNR2 expression, the expression of all OXPHOS-related genes was found to be reduced by M-LPH-KO (Figure 6(b)). Taken together, these findings strongly suggested that mitochondrial dysfunction due to mtDNA damage was prevented by an increase in mtDNA copy number, probably through a compensatory mechanism in HepG2 cells.

### 3.7. M-LPH-KO Changes the Intramitochondrial Protein Levels of TFAM, OGG1, LIG3, and TWNK

**Figure 9:**
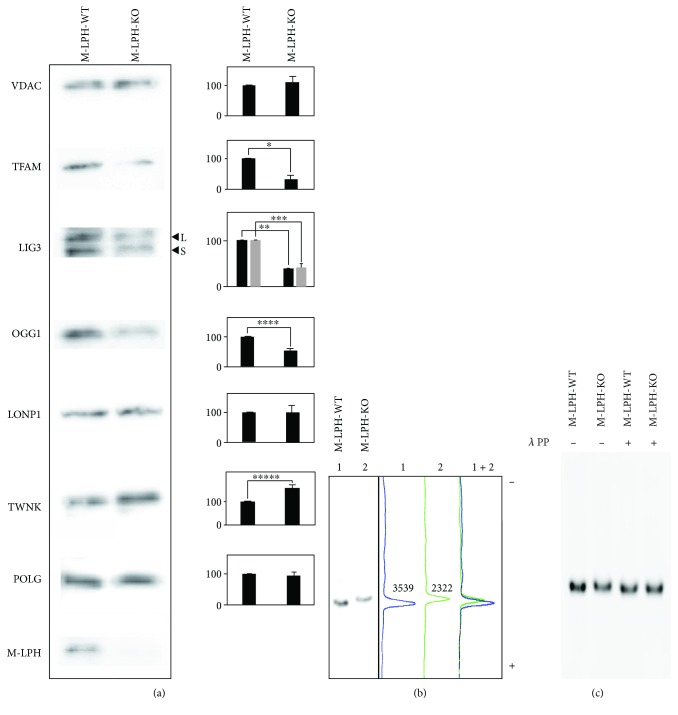
Western blot analysis of mitochondrial extracts from M-LPH-WT and -KO HepG2 cells. (a) The intramitochondrial levels of proteins essential for mtDNA stability and maintenance were examined using SDS-PAGE gels. Analysis was performed using the membrane extract (ME) used in Figure 2(b). VDAC was used as a loading control for mitochondrial protein. L and S in the figure correspond to the two mitochondrial isoforms of LIG3 generated by the alternative splicing. Three preparations of the membrane extract from WT and KO cells, respectively, were used for analysis. The bars represent the mean ± SD of the results from three independent experiments. Differences at *p* < 0.05 were considered to be statistically significant (^∗^*p* = 0.016, ^∗∗^*p* = 0.00018, ^∗∗∗^*p* = 0.0075, ^∗∗∗∗^*p* = 0.0091, and ^∗∗∗∗∗^*p* = 0.03). (b) Western blot analysis using Zn^2+^-Phos-tag gels was performed to investigate the state of phosphorylation of TFAM. In Zn^2+^-Phos-tag gel electrophoreses, phosphorylated proteins exhibit lower electrophoretic mobility than do corresponding dephosphorylated proteins [27]. In the presence of 50 *μ*M Phos-tag, the Rf values for TFAM band in M-LPH-KO cells were smaller than those in M-LPH-WT cells. Numbers in graphs represent the intensity of each band. (c) Mitochondrial extracts from M-LPH-WT and -KO cells were incubated with or without 40 U/*μ*l of *λ* protein phosphatase and analyzed by Zn^2+^-Phos-tag SDS-PAGE followed by immunoblotting with the anti-TFAM antibody.

**Figure 10:**
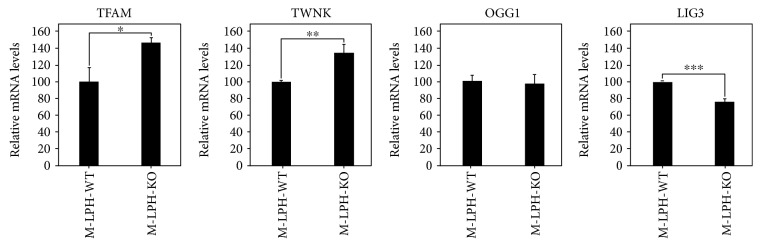
mRNA levels of the genes essential for mtDNA stability and maintenance. Intracellular expression of each gene was normalized against that of *β*-actin. The results are expressed as ratios relative to the value for M-LPH-WT cells. The bars represent the mean ± SD of the results from three independent experiments. Differences at *p* < 0.05 were considered to be statistically significant (^∗^*p* = 0.011, ^∗∗^*p* = 0.041, and ^∗∗∗^*p* = 0.00028).

In order to investigate the molecular mechanism responsible for the increases in damage and copy number of mtDNA, we examined the intramitochondrial levels of proteins essential for mtDNA stability and maintenance. Interestingly, M-LPH-KO resulted in obvious reductions in the protein levels of TFAM, LIG3, and OGG1 and an increase in that of TWNK (Figure 9(a)). Alterations of TFAM and TWNK levels in M-LPH-KO cells were also confirmed by confocal immunofluorescence (Figures 2(e) and 2(f)). In order to clarify whether this regulation occurred at the protein or RNA level, we examined the corresponding levels of expression of the mRNAs. As shown in Figure 10, the mRNA levels of TFAM and TWNK, which are essential components for mitochondrial transcription and replication, were significantly increased, whereas that of LIG3 was decreased, though the degree of decrease at the protein level (approx. 60%) was much higher than that at the mRNA level (23.4%). On the other hand, the level of OGG1 mRNA was unchanged. Accordingly, at least with regard to TFAM, LIG3, and OGG1, it appeared that the reductions were all attributable to posttranslational regulation, whereas TWNK was upregulated at both the mRNA and protein levels.

TFAM is known to be regulated through selective proteolysis by LONP1, the major protease involved in protein quality control in mitochondria. It is well known that the protein levels of LONP1 and TFAM show a strong inverse correlation [31]. However, under the experimental conditions we employed, we observed that the reduction of TFAM was not attributable to upregulation of LONP1 because its level was unchanged by M-LPH-KO (Figure 9(a)). Another possible explanation for the reduction of TFAM is that M-LPH-KO enhanced the phosphorylation of TFAM, as it has been proposed that phosphorylation impairs mtDNA binding of TFAM and that DNA-free TFAM is selectively degraded by LONP1 [31]. To investigate the state of phosphorylation of TFAM, we performed Western blot analysis using Zn^2+^-Phos-tag gels. On Zn^2+^-Phos-tag gels, the Rf values for all phosphorylated proteins are smaller than those for corresponding dephosphorylated proteins [27]. As we expected, the Rf value for the TFAM band in M-LPH-KO cells was smaller than that in M-LPH-WT cells (Figure 9(b)). Moreover, treatment with *λ* protein phosphatase increased the mobility of TFAM in M-LPH KO cells, whereas that from M-LPH-WT cells was unaltered (Figure 9(c)). Therefore, it was suggested that the phosphorylation status of TFAM was influenced (phosphorylation was promoted) by M-LPH deficiency.

## 4. Discussion

TFAM is a nuclear-encoded HMG family protein and the most abundant component of mitochondrial nucleoids. TFAM plays significant roles in transcription, replication, packaging, and organization of mtDNA. Under normal conditions, ~1000 molecules of TFAM are present per one molecule of mtDNA, thus fully covering mtDNA and increasing flexibility to promote compaction [31]. Mutations in this gene are associated with mtDNA depletion syndrome [32], and treatment with TFAM as an experimental therapy for Parkinson's disease has been suggested by data from a cellular model [33]. It has been extensively reported that KD or KO of TFAM decreases the mtDNA copy number and that the amounts of TFAM and mtDNA are strongly correlated [10, 34]. Based on the previous observation that TFAM did not upregulate mtDNA replication, TFAM was considered to maintain mtDNA by increasing its stability, serving as a protective shield [7, 9, 10, 31]. In the present study, we found that the protein level of TFAM was significantly reduced in M-LPH-KO cells (Figure 9(a)). This suggested that the increased degree of mtDNA damage observed in M-LPH-KO cells (Figures 4(a) and 5) was caused by instability of mtDNA associated with the reduction of TFAM. More interestingly, not only TFAM but also two DNA repair enzymes, OGG1 and LIG3, were reduced at the protein level by M-LPH-KO. OGG1 is responsible for the excision of 8-oxoguanine, which is commonly generated by ROS, while LIG3 functions as a DNA ligase. These enzymes are involved in mitochondrial BER, the most prominent mtDNA repair pathway, and located in both the mitochondria and nucleus. Taken together, these findings suggest that the increased degree of mtDNA damage in M-LPH-KO cells is probably attributable to two factors: instability of mtDNA caused by reduction of TFAM and a reduction of mtDNA damage repair capability resulting from lack of the two essential BER enzymes.

**Figure 11:**
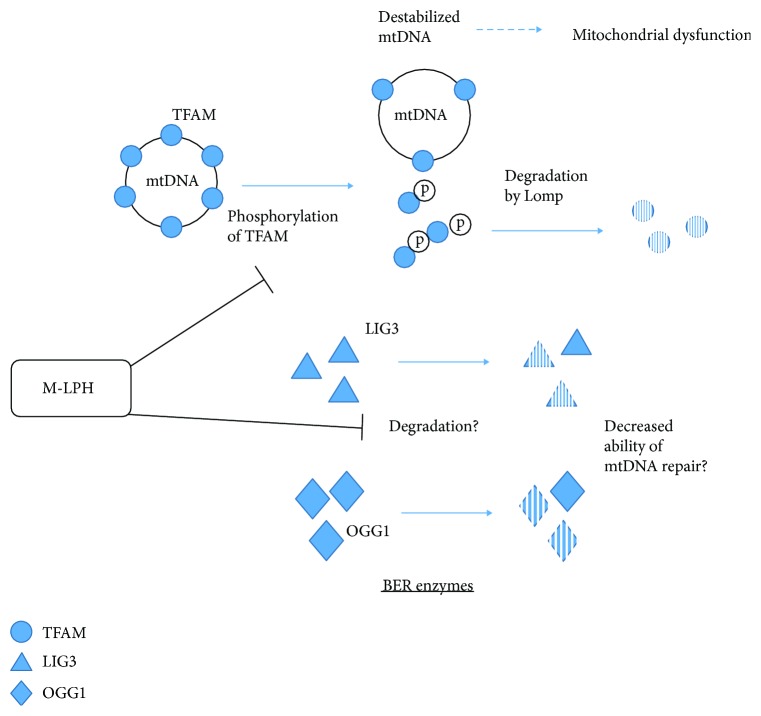
Mutual relationships among mtDNA, M-LPH, TFAM, and BER enzymes. TFAM fully coats mtDNA and protects it from oxidative damage. Phosphorylation of TFAM blocks its binding to mtDNA, and the DNA-free TFAM is rapidly and selectively degraded by LONP1. When the protective effect of TFAM is lost, mtDNA becomes vulnerable to oxidative stress, resulting in mitochondrial dysfunction. LIG3 and OGG1 are involved in mitochondrial BER. It is likely that M-LPH mitigates the phosphorylation of TFAM, thus protecting it from degradation.

In this study, we demonstrated that mitochondrial TFAM and OGG1, both of which are responsible for mtDNA integrity and maintenance, were regulated at the protein level. These results are consistent with a previous observation that the mRNA and protein levels of BER enzymes are correlated poorly with each other [35]. Expression of TFAM protein is regulated in several ways, including methylation of the TFAM gene promoter, inhibition of gene expression by miRNA [36], and selective degradation by LONP1. LONP1 is an ATP-dependent protease involved in degradation of misfolded, missorted, and damaged mitochondrial proteins and is known to be a component of mitochondrial nucleoids [31, 37]. It is also well known that the protein levels of LONP1 and TFAM show a strong inverse correlation [31]. However, in this study, we confirmed that the level of LONP1 was not affected by M-LPH-KO. Meanwhile, a recent study has proposed a phosphorylation-mediated regulation mechanism for TFAM: phosphorylation of TFAM at serines 55 and 56 by protein kinase A blocks its binding to mtDNA, and the resulting DNA-free TFAM is rapidly and selectively degraded by LONP1. Interestingly, our experimental results suggested that M-LPH exerted direct or indirect influences on the states of phosphorylation of TFAM and that the states of phosphorylation would be related to their susceptibility to degradation. In other words, M-LPH could be involved in the maintenance of mtDNA, and therefore mitochondrial function, by protecting the proteins essential for mtDNA stability and maintenance from degradation in an integrated manner (Figure 11).

In the present study using M-LPH-KO HepG2 cells, we observed an increase in the copy number of mtDNA concomitant with an increase in mtDNA damage (Figure 8). Among the proteins involved in mtDNA integrity, only TFAM and TWNK have been shown to correlate significantly with mtDNA copy number. Homozygous KO of TFAM or TWNK are both lethal in mice, and mice with heterozygous KO of either exhibit a reduction in mtDNA copy number [38, 39]. On the other hand, transgenic mice overexpressing TFAM or TWNK show a 1.5- to 2- [40, 41] and 2- to 3-fold [41, 42] increase in mtDNA copy number, respectively. Although the effects on mtDNA copy number caused by disruption of the TFAM or TWNK gene are remarkably similar to each other, the mechanisms responsible for the mtDNA copy number regulation are considered to be quite different: TFAM works by increasing the packaging and stability of mtDNA, whereas TWNK works by upregulating its replication [43]. In the present study, M-LPH-KO yielded an increase in TWNK simultaneously with a decrease in TFAM at the protein level (Figure 9(a)). Therefore, the mtDNA copy number increase observed in M-LPH-KO HepG2 cells is likely caused by the increase in TWNK and resulting upregulation of replication.

In our previous study with human normal kidney HK-2 cells, we observed that siRNA-mediated knockdown of M-LPH caused an increase in ROS generation and loss of mitochondrial ΔΨ_m_ in addition to mtDNA damage [20]. By contrast, in the present study using M-LPH-KO HepG2 cells, no significant influence on ΔΨ_m_ or ROS and ATP levels was detected, despite the increase in mtDNA damage (Figure 7). We assume that these differences between HK-2 and HepG2 cells were due to the existence or lack of a compensatory mechanism for increasing the mtDNA copy number in response to mtDNA damage. In fact, in our previous study, it was confirmed that siRNA-mediated knockdown of M-LPH had no influence on the mtDNA content of HK-2 cells [20], normal human renal proximal tubule epithelial cells (RPTEC), and human breast cancer cells (MDA-MB-453) (R. Iida, M. Ueki, and T. Yasuda, unpublished data). Moreover, the following observation can also be considered to provide additional evidence: before the generation of M-LPH-KO HepG2 cells, we first tried to generate M-LPH-KO HK-2 cells, but all of the single-cell clones did not proliferate and gradually died within two months. Therefore, it seems likely that the present successful generation of KO cells was probably due to the particular characteristics of HepG2 cells. Differences in cellular responses to mtDNA damage such as this may be one of the elements to consider when devising therapeutic approaches for diseases associated with mitochondrial dysfunction caused by mtDNA damage, including cancer, neurodegenerative disorders, and cardiovascular diseases.

Here, we focused on how M-LPH protects mtDNA from oxidative damage in HepG2 cells. In addition to mtDNA damage, it seems very likely that M-LPH also plays a role in protection of nDNA from damage, as firstly not only mtDNA damage but also nDNA damage were increased by M-LPH-KO HepG2 cells, and secondly it has been shown previously that M-LPH interacts with H2AX [20], which is closely correlated with the nDNA damage response. The molecular function of M-LPH in the protection of nDNA is currently under investigation.

## Figures and Tables

**Table 1:** Primers used for Q-PCR analysis.

Gene/region	Forward primer (5′-3′)	Reverse primer (5′-3′)
D-Loop		
368–476	ACCCTAACACCAGCCTAACCA	GTAGTATGGGAGTGGGAGGGGA
16,457–16,535	GCCCATAACACTTGGGGGTA	TTTAAGGGGAACGTGTGGGC
TFAM	GCTTGGAAAACCAAAAAGACCTCGT	CGACGTAGAAGATCCTTTCGTCCAA
TWNK	AGCCAAAGCAAGCCAGGAA	AAAGCGGTTCTTGGACACCT
SDHA	ACCAACTACAAGGGGCAGGT	CGACCAAAGACAACCAGGTC
COX7B	AGCCACCAGAAACGTACACC	TGGGGTAACTCTGCCAACA
ATP50	CGTTTCTCTCTTCCCACTCG	GTGGCATAGCGACCTTCAAT

## Data Availability

No data were used to support this study.
